# The effect of insecticide synergist treatment on genome-wide gene expression in a polyphagous pest

**DOI:** 10.1038/s41598-017-13397-x

**Published:** 2017-10-18

**Authors:** Simon Snoeck, Robert Greenhalgh, Luc Tirry, Richard M. Clark, Thomas Van Leeuwen, Wannes Dermauw

**Affiliations:** 1 Laboratory of Agrozoology, Department of Crop Protection, Faculty of Bioscience Engineering, Ghent University, Coupure links 653, 9000 Ghent, Belgium; 2 Department of Biology, University of Utah, 257 South 1400 East, Salt Lake City, Utah 84112 USA; 3 Center for Cell and Genome Science, University of Utah, 257 South 1400 East, Salt Lake City, Utah 84112 USA; 4 Institute for Biodiversity and Ecosystem Dynamics (IBED), University of Amsterdam (UvA), Science Park 904, 1908 XH Amsterdam, The Netherlands

## Abstract

Synergists can counteract metabolic insecticide resistance by inhibiting detoxification enzymes or transporters. They are used in commercial formulations of insecticides, but are also frequently used in the elucidation of resistance mechanisms. However, the effect of synergists on genome-wide transcription in arthropods is poorly understood. In this study we used Illumina RNA-sequencing to investigate genome-wide transcriptional responses in an acaricide resistant strain of the spider mite *Tetranychus urticae* upon exposure to synergists such as S,S,S-tributyl phosphorotrithioate (DEF), diethyl maleate (DEM), piperonyl butoxide (PBO) and cyclosporin A (CsA). Exposure to PBO and DEF resulted in a broad transcriptional response and about one third of the differentially expressed genes (DEGs), including cytochrome P450 monooxygenases and UDP-glycosyltransferases, was shared between both treatments, suggesting common transcriptional regulation. Moreover, both DEF and PBO induced genes that are strongly implicated in acaricide resistance in the respective strain. In contrast, CsA treatment mainly resulted in downregulation of Major Facilitator Superfamily (MFS) genes, while DEGs of the DEM treatment were not significantly enriched for any GO-terms.

## Introduction

Insecticide resistance is a major threat for the agricultural productivity of commercial crops^1^, and understanding the mechanisms underlying insecticide resistance is a high priority for the design and implementation of effective resistance management programs^2^. Resistance mechanisms can generally be classified into either (1) changes in sensitivity of the target-site due to point mutations, or to (2) increased metabolic detoxification through qualitative or quantitative changes in enzymes involved in the detoxification process. The latter process typically occurs in 3 phases. In phase I the insecticide is functionalized with nucleophilic groups (a hydroxyl, carboxyl or amine group) to make it more reactive and water soluble. In phase II, conjugation occurs with endogenous molecules (such as glutathione (GSH) or sugars), further increasing the compound’s polarity. Ultimately, in phase III, the phase II conjugated product is excreted by cellular transporters. Cytochrome P450 monooxygenases (P450s) and carboxyl/choline esterases (CCEs) are well-known examples of enzymes that are responsible for phase I reactions while glutathione-S-transferases (GSTs) and UDP-glycosyltransferases (UGTs) are enzymes that typically operate during phase II. Finally, in phase III, metabolites are often transported out of the cell by ATP-binding cassette (ABC) transporters and solute carrier proteins, of which a major class are proteins of the Major Facilitator Superfamily (MFS)^3,4^.

Insecticide synergists are defined as “compounds that greatly enhance the toxicity of an insecticide, although they are usually practically nontoxic on their own”^5^. They can either act as a surrogate substrate or an inhibitor of detoxification enzymes and transporters and, as such, are a powerful tool to investigate insecticide resistance mechanisms. Synergists are also of commercial interest as combining them with insecticides increases efficacy and aids in keeping pesticide use to a minimum^6–8^. As a result of the fast and widespread development of resistance, coupled with the slowdown in the number of registrations of new pesticides and a new trend towards “greener” and more “sustainable” pest management^9^, a renewed interest has arisen in the identification and development of plant-based synergists^10–12^. However, as of yet relatively few of these new synergists have made the transition from the laboratory to the field or greenhouse.

The most well-known and commonly used commercial insecticide synergist is the methylene dioxyphenyl compound piperonyl butoxide (PBO), an inhibitor of P450s. PBO has been commercially used since 1940, mainly in combination with pyrethroid insecticides. Its lack of specificity in P450 inhibition has contributed to its success as a synergist^13^. The inhibition mechanism of PBO consists of two phases, starting with the binding of PBO to the active site of the P450, followed by the formation of a quasi-irreversible inhibitor complex between the electrophilic carbene moiety of PBO and the ferrous iron of the P450. This results in a decreased metabolic activity of the P450 enzyme^7,14,15^. Synergists such as the defoliant S,S,S-tributyl phosphorotrithioate (also known as tribufos, DEF or TBPT), the fungicide iprobenfos (IBP) and triphenyl phosphate (TPP) are well-known carboxyl esterase inhibitors^16,17^. These organophosphorus compounds (OPs) behave like the natural substrate of esterases and enter the active site where they covalently bind to the serine –OH group. Subsequently, the OP is split, with the enzyme being non–reversibly phosphorylated, and regeneration of the free enzyme by hydrolyzation not possible^18,19^. An additional major synergist is the carbonyl compound diethyl maleate (DEM) that is known to conjugate reduced glutathione (GSH), thereby depleting cells of this tripeptide. As a consequence, it reduces the ability of GSTs to utilize GSH for conjugation with insecticides or with the oxidative stress products they induce^20–22^. Finally, verapamil and cyclosporin A are well-known first generation modulators (competitive inhibitors) of vertebrate P-glycoproteins (ABC transporters of the B subfamily)^23–25^. Human P-glycoproteins are well-known for their role in protecting tissues from toxic xenobiotics and endogenous metabolites^26^ and in the last decade their counterparts in arthropods have also been linked to insecticide transport and/or resistance^27,28^. For example, pretreatment with verapamil has been shown to markedly enhance the toxicity of DDT or abamectin in *Drosophila* ^29,30^.

Synergists, however, do not always act as intended or expected. A frequently reported unanticipated effect is an altered cuticular penetration of the insecticide after pretreatment with a synergist^31–34^. In some cases, synergists might also inhibit non-target enzyme systems. PBO has been reported to act as an inhibitor of esterases in *Helicoverpa armigera*, *Frankliniella occidentalis* and *Bemisia tabaci* ^35–37^ and as an inhibitor of mammalian UGTs^38^, while DEF was shown to act, albeit to a much lower extent compared to PBO, as an inhibitor of P450s in *Blatella germanica* ^34^. These studies suggest that in some cases caution should be applied in interpreting results of synergist application as they are not entirely specific to a single detoxification enzyme class^37,39,40^. However, inhibition of these non-target enzyme systems typically occur in a non-specific way at high synergist concentrations^41,42^, and results can be cross-checked. For example, confirmation of P450 rather than esterase inhibition by PBO is straightforward and can be done with another class of P450 inhibitors^13^.

Insects and mites are known to display a massive and rapid reprogramming of gene expression in response to xenobiotic exposure^43–45^. While synergist compounds are mostly used at concentrations that cause no or very low mortality, they are nonetheless used at high enough concentrations to cause maximal inhibition of the targeted detoxification enzymes^46^. Hence, one might expect that synergists also induce gene expression changes on their own^6^. Little is known, however, regarding the genome-wide transcriptional changes in arthropods exposed to synergists. Using a full genome and a custom “detox” microarray it was found that a subset of P450s and GSTs, and to a lesser extent UGTs, were induced in *Drosophila melanogaster* upon exposure to PBO^47^, while, using Illumina RNA sequencing, a P450 was shown to be upregulated in the whitefly *B. tabaci* upon exposure to PBO + cypermethrin as compared to cypermethrin alone^48^. However, genome-wide transcriptional changes upon exposure to synergist compounds other than PBO have not been investigated in any herbivorous arthropod pest.

The two-spotted spider mite, *Tetranychus urticae* (Arthropoda: Chelicerata: Acari: Tetranychidae), is a highly polyphagous agricultural pest that is able to colonize more than 1100 plant species^49^. Further, among arthropods *T. urticae* is also considered to be the ‘‘pesticide resistance champion” based on the total number of different pesticides to which populations have become resistant^50,51^. Synergists such as PBO, DEF and DEM have been frequently used for the identification of metabolic resistance pathways in *T. urticae* ^52–61^, and a high quality Sanger genome assembly is available for this mite species^62^. *T. urticae* is therefore an ideal arthropod herbivore to study the impact of synergists at the transcriptional level. In this study, we used Illumina HiSeq 2500 technology to generate deep paired-end, strand-specific RNA-seq reads from adult *T. urticae* females that were exposed for 24 hours to either DEF, DEM, PBO, CsA or formulation only. Subsequent differential expression (DE) analyses allowed us to identify genes for which expression was significantly altered upon exposure to synergists. A selection of differentially expressed genes was validated by qPCR and for each comparison (synergist compared to formulation) a Gene Ontology (GO) analysis was performed to shed light on the types of genes and pathways that respond to synergist exposure (DEGs).

## Results

### Synergist bioassays and RNA-seq

Adult female mites of the JP-R strain were either not sprayed (CON) or sprayed with PBO (1000 ppm), DEF (500 ppm), DEM (2000 ppm), CsA (50ppm) or formulation (FORM; N,N-dimethylformamide and emulsifier W (3:1 w/w) diluted in deionized water 100-fold) and collected after 24 hours, a commonly used time point in synergist studies^56,63–65^. Mortality for each treatment was scored, and found to be in line with those of previous reports for that strain^56^. Mites alive at 24 hours were collected for each treatment (PBO, DEF, DEM, CsA) and the controls (CON, FORM) and used for RNA extraction and RNA-seq library generation. Illumina sequencing generated ~82–92 million strand-specific paired end reads per sample. Alignment of RNA-seq reads against the *T. urticae* annotation resulted in an overall mapping rate of uniquely mapped reads of 83.01 ± 0.19 SE% across samples (Supplementary Table S1).

### Principal Component Analysis (PCA)

**Figure 1:**
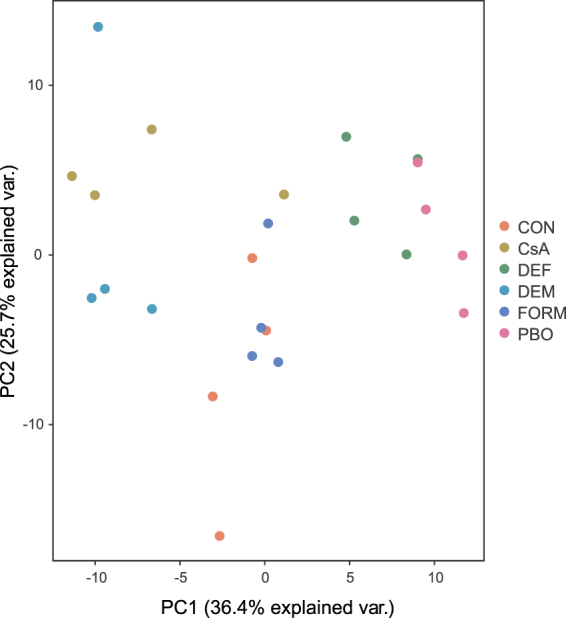
Gene expression relationships among synergist treatments and controls. PCA plot of gene expression levels in untreated adult *T. urticae* females (CON), adult *T. urticae* females sprayed with formulation only (FORM) or adult *T. urticae* females exposed to synergist compounds CsA, DEF, DEM or PBO.

A PCA revealed that about forty percent (36.8%) of the total variation could be explained by PC1 while PC2 accounted for 25.7% of the variation (Fig. 1). Except for replicate 4 of the CsA treatment, biological replicates clustered by treatment on PC1 relatively well. On the other hand, all replicates of the CsA treatment clustered relatively well on PC2. Interestingly, in the PCA the expression data of PBO treatments clustered most closely with those of the DEF treatments, those of the FORM treatments grouped with those of unsprayed mites (CON), and those of the CsA treatments grouped with those of the DEM treatments.

### Differential expression analysis and qPCR validation

**Figure 2:**
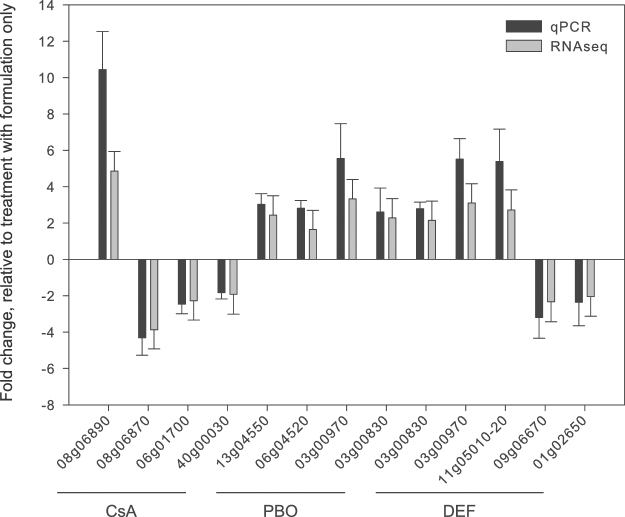
qPCR validation of differentially expressed genes in adult *T. urticae* females after PBO, DEF or CsA treatment. Eight up- and five downregulated genes as assessed by differential gene expression (DESeq2) analysis of RNA-seq data were selected for qPCR analysis. Error bars represent the standard error of the calculated mean. Except for *tetur40g00030* (CsA treatment), each gene was significantly differentially expressed (based on an unpaired t-test) compared to the reference condition (FORM). The ‘‘tetur’’ prefix was removed from *T. urticae* gene IDs for figure clarity.

**Figure 3:**
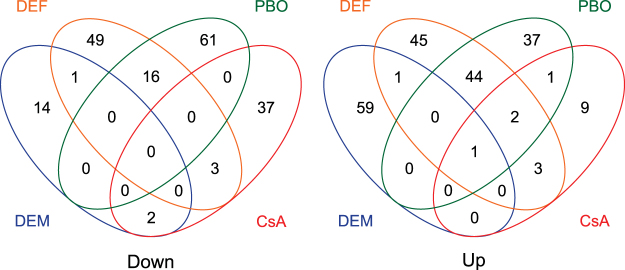
Venn diagrams depicting overlap among differentially expressed genes of adult *T. urticae* females exposed to either PBO, DEF, DEM or CsA compared to adult *T. urticae* females treated with formulation only. Left panel: downregulated genes, right panel: upregulated genes.

We used DESeq2 to perform differential gene expression (DEG) analyses between treatments and controls (fold change (FC) ≥ 1.5 and a Benjamini-Hochberg adjusted p-value < 0.05)^66^. Between mites sprayed with formulation and unsprayed mites, only one gene (*tetur13g00990*, coding for an “orphan secreted protein”) was differentially expressed (FC of 1.53), indicating that the formulation on its own had no significant effect on gene expression. Next, we compared gene expression levels between mites treated with one of the synergists and mites that were treated with formulation only. One hundred and sixty-two genes were differentially expressed between mites treated with PBO and mites treated with formulation, of which 77 were downregulated and 85 upregulated (Supplementary Table S2). The top twenty down- and upregulated genes had a FC of −2.11 to −1.74 and 1.86 to 3.33 respectively. For the DEF treatment, 174 genes were differentially expressed, of which 69 were downregulated and 105 were upregulated (Supplementary Table S3). The expression level of the 20 most down- and upregulated genes varied from a FC of −2.33 to −1.73 and 1.88 to 3.10, respectively. For the DEM treatment, 78 genes were differentially expressed, with 17 genes being downregulated and 61 upregulated and the expression level of the 20 most down- and upregulated genes varied from a FC of −2.3 to −1.5 and 1.5. to 2.1, respectively (Supplementary Table S4). Finally, for the CsA treatment, 58 genes were differentially expressed. Forty-two of them were downregulated, while 16 were upregulated and the expression level of the 20 most downregulated genes varied from −3.87 to −1.72 (Supplementary Table S5). For a selection of genes, the differential expression analyses based on RNA-seq were consistent with differential expression as validated independently by qPCR (Fig. 2). As shown in Fig. 3, the majority of DEGs were not shared between the different treatments. Only one upregulated gene was in common for all treatments (*tetur01g06580*, a sodium dependent glucose transporter), while two upregulated genes (*tetur16g03490* and *tetur11g05520*, coding for an antigen B membrane protein and CYP385C4, respectively) were shared between the PBO, DEF and CsA treatments. In contrast, 60 DEGs (37% and 35% of the total number of DEGs for the PBO and DEF treatment, respectively) were shared between PBO and the DEF treatment and had the same direction of fold change, with 44 genes being upregulated and 16 being downregulated.

### GO enrichment and cluster analysis

**Figure 4:**
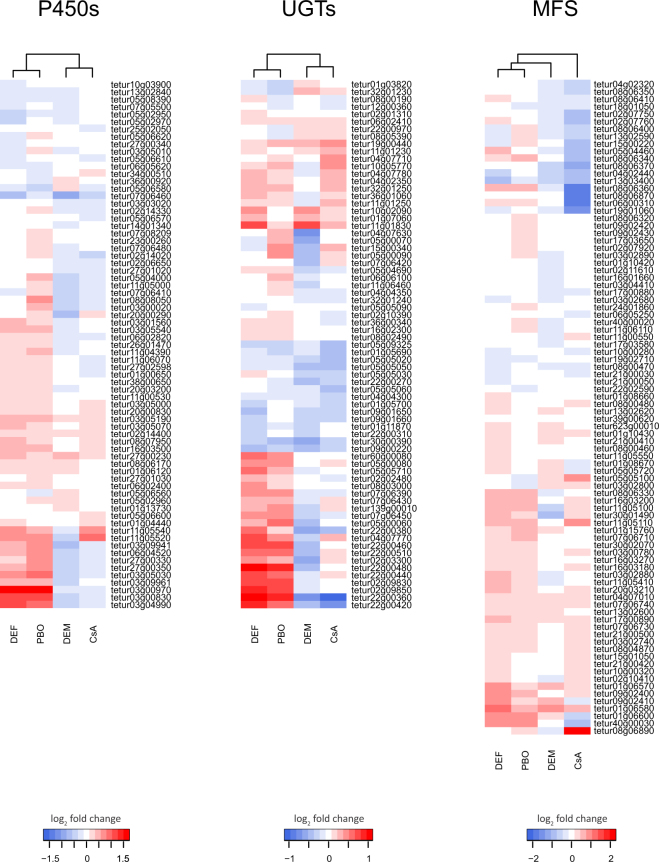
Expression heatmaps of genes coding for P450s, UGTs or MFS members in adult *T. urticae* females exposed to either DEF, PBO, DEM or CsA. The log_2_ transformed fold changes are relative to adult *T. urticae* females treated with formulation only and were clustered using a Euclidean distance metric and Ward’s method. *T. urticae* gene IDs are shown on the right.

We next performed GO enrichment analyses for the various differentially expressed gene sets. For the DEGs of the DEM treatment, no significantly (Benjamini-Hochberg adjusted p-value < 0.05) enriched GO terms were identified, while for the CsA treatment DEGs were significantly enriched in the GO terms “transmembrane transport” (GO:0055085) and “integral component of membrane” (GO:0016021) (Table 1). Inspecting the DEGs in more detail revealed that these GO terms were mainly present in genes coding for transporters of the major facilitator superfamily (MFS, InterPro domain IPR020846) (20 out of 58 DEGS, 34.5%), and the majority of these MFS genes (15 out of 20) were downregulated (Supplementary Table S5, Supplementary Table S6, Fig. 4). Based on their best BLASTp hit in the Transporter Classification database^67^, these MFS genes belong to either the Oxalate:Formate Antiporter Family (1/20), Proton Coupled Folate Transporter/Heme Carrier Protein Family (4/20), Fucose H + Symporter (5/20) or the Anion/Cation Symporter (10/20) MFS subfamily. The GO:0016021 term was also associated with genes coding for P450s (3 DEGs), UGTs (2 DEGs), cation-proton exchanger proteins (InterPro domain IPR018422, 2 DEGs) and a protein with an Apple-like domain (tetur25g02030, InterPro domain IPR003609). Interestingly, genes coding for ABC transporters, which are known to be inhibited by CsA^68^, were not found among the DEGs of the CsA treatment.

**Table 1:** GO enrichment analysis of differentially expressed genes (absolute fold change ≥1.5 and Benjamini-Hochberg adjusted p-value < 0.05) in adult *T. urticae* females of the JP-R strain exposed to either PBO, DEF or CsA compared to adult *T. urticae* females treated with formulation only.

Treatment	GO Category	Description	adjusted p-value	Subontology*
CsA	GO:0055085	transmembrane transport	0	BP
CsA	GO:0016021	integral component of membrane	6.72^E^-05	CC
PBO	GO:0016705	oxidoreductase activity, acting on paired donors, with incorporation or reduction of molecular oxygen	2.68^E^-09	MF
PBO	GO:0020037	heme binding	3.05^E^-08	MF
PBO	GO:0004497	monooxygenase activity	3.05^E^-08	MF
PBO	GO:0005506	iron ion binding	3.08^E^-08	MF
PBO	GO:0055114	oxidation-reduction process	1.28^E^-05	BP
PBO	GO:0016999	antibiotic metabolic process	8.83^E^-04	BP
PBO	GO:0016758	transferase activity, transferring hexosyl groups	2.21^E^-02	MF
DEF	GO:0016758	transferase activity, transferring hexosyl groups	2.86^E^-05	MF
DEF	GO:0016999	antibiotic metabolic process	4.00^E^-05	BP
DEF	GO:0005506	iron ion binding	1.45^E^-04	MF
DEF	GO:0055114	oxidation-reduction process	3.43^E^-04	BP
DEF	GO:0020037	heme binding	4.93^E^-04	MF
DEF	GO:0016705	oxidoreductase activity, acting on paired donors, with incorporation or reduction of molecular oxygen	5.10^E^-04	MF
DEF	GO:0004497	monooxygenase activity	7.96^E^-04	MF
DEF	GO:0005179	hormone activity	1.18^E^-03	MF
DEF	GO:0016021	integral component of membrane	1.66^E^-03	CC

*BP, Biological Process; MF, Molecular Function; CC, Cellular Component.

DEGs of both the PBO and DEF treatment were significantly enriched in the GO terms “oxidoreductase activity, acting on paired donors, with incorporation or reduction of molecular oxygen” (GO:0016705), “heme binding” (GO:0020037), “monooxygenase activity” (GO:0004497), “iron ion binding” (GO:0005506), “oxidation-reduction process” (GO:0055114), “antibiotic metabolic process” (GO:0016999) and “transferase activity, transferring hexosyl groups” (GO:0016758). The first five GO terms were mainly present in genes coding for P450s (14 and 11 DEGs for the PBO and DEF treatments, respectively) while the latter two terms were found in UGTs (7 and 12 DEGs for the PBO and DEF treatments, respectively). Interestingly, almost all P450 and UGT genes (13/14 P450s and 7/7 UGTs (PBO treatment) and 10/11 P450s and 12/12 UGTs (DEF treatment)) were upregulated for both the PBO and DEF treatment. The majority of the upregulated P450 genes (12/13 and 8/10 for the PBO and DEF treatments, respectively) belonged to the CYP392 family within the CYP2 clan for which several members were shown previously to respond strongly to acaricide selection and feeding on different hosts^44^. GO:0055114 was also found in genes coding for short chain dehydrogenases (4 and 3 DEGs for the PBO and DEF treatments, respectively) and intradiol ring-cleavage dioxygenases (2 and 4 DEGs for the PBO and DEF treatments, respectively) while for the DEF treatment this term was also present in genes coding for a superoxide dismutase (*tetur02g11180*), a fatty acid synthase (*tetur04g02370*) and a glucose dehydrogenase (*tetur03g09330*). In addition, DEGs of the DEF treatment were also significantly enriched in the GO terms “hormone activity” (GO:0005179) and “integral component of membrane” (GO:0016021). The “hormone activity” term was present in four DEGs coding for neuropeptides (InterPro domain IPR001955 or IPR016179), while “integral component of membrane” was present in more than 45 DEGs, including, amongst others, genes coding for P450s (10 DEGs), MFS proteins (10 DEGs), UGTs (7 DEGs), ABC transporters (3 DEGs), neuropeptides (2 DEGs), innexins (InterPro domain IPR000990, 2 DEGs) and proteins with a DUF3421 domain (InterPro domain IPR024518, 2 DEGs) (Table 1 and Supplementary Tables S2 and S3). A cluster analysis of expression data of P450, UGT and MFS genes complemented the findings of our GO analyses. For both the P450 and UGT gene expression data the PBO and DEF treatment clustered together, while for MFS gene expression data, the CsA treatment fell outside the clade with all other treatments (Fig. 4).

## Discussion

Gene expression changes are a major component of stress responses, and contribute to or complement alterations in metabolism, cell cycle progression, protein homeostasis, cytoskeletal organization, vesicular trafficking and modification of enzymatic activities^69^. Expression changes can be grouped into generic responses shared by many stresses, and stress-specific adaptive responses. In contrast to post-translational effects, which provide immediate responses, regulation of gene expression is essential for the slower, long term acclimation to and recovery from a stress factor^69^. At present, our understanding of gene expression changes in arthropods to synergist exposure is very limited in spite of their importance in insecticide- and acaricide-mediated control. In fact, only two studies have investigated genome-wide transcriptional changes in an insect upon synergist exposure. Willoughby *et al*. and Zimmer *et al*.^47,48^ studied PBO-induced gene expression changes in *D. melanogaster* and *B. tabaci*, respectively. Gene expression changes upon exposure to other synergist compounds have not yet been determined.

In this study, we examined transcriptional responses in a polyphagous arthropod pest, *T. urticae*, upon exposure to the synergist compounds PBO, DEF, DEM and CsA. The strain that was used for our experiments (JP-R, see Material & Methods) was shown to be resistant to the mitochondrial complex II inhibitors cyenopyrafen and cyflumetofen (both compounds are beta-keto nitrile derivatives belonging to IRAC Mode of Action Classification 25 A^ 2,70^), with cyenopyrafen and cyflumetofen toxicity strongly synergized by PBO and DEM, respectively, while DEF moderately synergized cyenopyrafen toxicity^64^. Because acaricides are typically applied 24 h after synergist treatment^53,56,64,65^, transcriptional changes were captured 24 h after exposure. In future, it would be interesting to also capture the transcription levels at those synergist exposure periods that have been used in previous synergism studies with *T. urticae* (1 h and 4 h)^53,55,57–61^. Finally, the observed fold changes of up- and downregulated *T. urticae* genes (ranging from −3.8 to 3.3 across all synergist treatments) were in line with those observed for *B. tabaci* by Zimmer *et al*.^48^. In contrast, fold changes of PBO induced *Drosophila* genes were considerably higher (ranging from 2 to 32)^47^. However, in our study a 24 h synergist pretreatment was used, while in *Drosophila* expression measurements were taken after 4 hours of synergist exposure, and with the *Drosophila* 16 K full genome array. Briefly, the *Drosophila* 16 K full genome array contained only one 70mer probe per gene^47^, confounding reliable estimation of fold changes^71^, while in our study and that of Zimmer *et al*.^48^ gene-expression levels were captured by Illumina RNA-sequencing.

Stress responses can be induced by a variety of stressors such as extreme temperatures, elevated ion concentrations or toxic substances, all of which usually result in excessive amounts of denatured proteins^72^. The general stress response typically consists of an increased production or activation of antioxidant proteins (e.g. Cu/Zn superoxide dismutase and GSTs) and chaperone proteins (e.g. heat shock proteins)^73,74^, together with an increased mobilization of energy from storage tissues^75^. The latter is associated with the overexpression of numerous genes involved in energy production or in cellular catabolism such as NADH dehydrogenase, ATP synthase, trypsin and lipases^76^. Only one GST gene was significantly upregulated in the PBO and DEF treatment while a gene coding for a superoxide dismutase was downregulated in the DEF treatment. A GO enrichment analysis did not reveal a GO category that was in common between the different synergist treatments and that was related to the above-mentioned proteins. Even more, only one upregulated gene, coding for a putative sodium-dependent glucose transporter (*tetur01g06580*, member of the MFS-gene family), was in common for all treatments. Hence, we assume that the observed gene expression changes are not general stress responses but rather specific for the synergist compounds to which the mites had been exposed. However, based on PC1 of our principal component analysis gene expression levels of PBO and DEF treatment replicates did cluster together (Fig. 1). Even more, about 35% of the *T. urticae* DEGs that were identified for both these treatments were shared and had fold changes in the same direction (Fig. 3). Interestingly, about one third (15/44) of the upregulated shared DEGs coded for members of the P450 and UGT gene families (Fig. 4, Supplementary Table S2). For *D. melanogaster*, it has been shown that the transcription factor CncC plays an important role in xenobiotic-inducible gene expression of P450s, GSTs, UGTs and membrane transporters^77^. However, none of the *T. urticae* orthologs of *D. melanogaster CncC* (*tetur07g06850* and *tetur07g04600* ^44^) were differentially expressed after 24 h (Supplementary Tables S2,S3,S4 and S5). In addition, *T. urticae* genes related to the gene coding for *Drosophila* Keap1 (Kelch-associated protein 1), a negative regulator of CncC, and that are downregulated in acaricide resistant and host plant adapted mite lines (OrthoMCL group 10254)^44,45^ were also not found among the DEGs of both the PBO and DEF treatments. However, expression changes of *T. urticae* transcription factors genes^78^ did correlate for these treatments (Supplementary Figure S1), suggesting a common transcriptional regulation. Ten of these transcription factor genes had a fold change of more than 1.25 in both the PBO and DEF treatments and had an opposite direction of fold change compared to the CsA and DEM treatments (Supplementary Table S7) and, hence, might be good candidates to be investigated for their role in xenobiotic resistance regulation in *T. urticae*.

The induction of P450s upon exposure by PBO has been reported by other groups^47,48,79–82^. As hypothesized by Chan *et al*.^81^, it might be a compensation strategy for an enzyme to be upregulated upon contact with an inhibitor in order to minimize the effect of reduced enzyme activity. In general, several studies have shown that xenobiotics are able to induce expression of arthropod P450s^11,44,45,48,77,83–86^. In *Drosophila* for example, about a third of the P450s can be induced following xenobiotic substance feeding or topical application^87^. For *T. urticae* it has been shown that the application of barbital, phenobarbital and geraniol does increase P450 activity in a dose-dependent way and that pretreatment with these inducers resulted in decreased acaricide toxicity^88^. Willoughby *et al*. suggested that the increase of detoxification enzymes by PBO might speed up insecticide tolerance if these detoxification enzymes could metabolize insecticides. Hence, one might question whether induction of *T. urticae* detoxification genes by synergist compounds could lead to a decreased toxicity of acaricides. In this study, *CYP392A11* and *CYP392A12* were both upregulated upon exposure of the JP-R strain by PBO. In 2015 it was shown that the upregulation of these genes was associated with cyenopyrafen resistance of the JP-R strain^64^ and that CYP392A11 could metabolize cyenopyrafen^89^. Similarly, only three carboxyl/choline esterases, *TuCCE06* (*tetur01g10800*), *TuCCE07* (*tetur01g010810*) and *TuCCE25* (*tetur04g06670*), were upregulated upon exposure of the JP-R strain by DEF and recently it was shown that TuCCE25 was highly upregulated in the JP-R strain and responded to cyenopyrafen selection^64^. In summary, both PBO and DEF seem to induce genes coding for detoxification enzymes that are strongly associated/implicated in cyenopyrafen resistance of the JP-R strain. On the other hand, both PBO and DEF, at the same concentration and exposure period that was used in this study, have previously been shown to synergize cyenopyrafen toxicity^64^. Hence, the direct inhibition of these P450s and CCEs by PBO and DEF, respectively, seems to outweigh the impact of the synergists on their induction. In contrast, in instances where P450s or CCEs may not be involved in acaricide resistance, PBO or DEF induction of genes encoding enzymes that are not inhibited by the synergist compound and that are capable of metabolizing acaricides might interfere and lead to misinterpretation of synergism experiments (for example antagonism instead of synergism).

Both the PBO and DEF treatments resulted in an upregulation of *TuGSTd14* (*tetur29g00220*). Of particular note, *TuGSTd14* was also upregulated in multi-resistant *T. urticae* strains^44^ and showed affinity toward abamectin and a competitive type of inhibition^90^. In contrast, the DEM treatment did not result in the overexpression of GSTs. Even more, significantly enriched GO terms could not be identified for the DEM treatment. This result was somewhat unanticipated as it is known that depletion of GSH by DEM results in oxidative stress and destabilized proteins *in vitro* ^91^ and that the response to oxidative stress typically consists of highly coordinated changes in gene expression (for example, in human carcinoma cells^92^). In the study by Casey *et al*.^92^, exposure to DEM resulted in GSH depletion below control levels from 1 h to 24 h after DEM treatment. However, although the expression of about 800 genes had a significant change (over two-fold at more than one time point) during this 24 h time course experiment, log_2_ fold changes of almost all genes returned to near zero at the 24 h time point^92^. Previously it was shown that a 24 h treatment with DEM strongly synergized cyflumetofen toxicity in the JP-R strain^64^ and that a GST (TuGSTd05) that was upregulated in this strain could metabolize cyflumetofen. Hence, the 24 h DEM exposure most likely resulted in depleted GSH levels in mites of the JP-R strain but the expression changes that are associated with the onset of depletion may not have been captured in our study.

The Major Facilitator Superfamily (MFS) is, along with ATP-binding cassette (ABC) transporters, one of the largest transporter families present in all living organisms. MFS transporters, as opposed to ABC transporters, use an existing electrochemical gradient instead of ATP to transport substrates (either by symport, uniport or antiport) across membranes^27,93^. Numerous studies have pointed to the importance of ABC transporters in xenobiotic resistance in arthropods^27,28^. In the case of *T. urticae*, several of its 103 ABC transporter genes were differentially expressed in multi-pesticide resistant strains and/or in mites transferred to a more challenging host plant^94^. MFS transporters, however, have been less studied in arthropods and it is only recently that evidence for their role in xenobiotic resistance in arthropods has been reported^44,95^. Consistent with this, 37 *T. urticae* MFS genes (belonging to either OrthoMCL group arthro10032, arthro10082 or arthro10236) were differentially expressed upon long term transfer of mites from bean to tomato^44^.

None of the 103 ABC *T. urticae* transporter genes were differentially expressed upon exposure to the ABC transporter modulator CsA. In contrast, about one third of the DEGS of the CsA treatment coded for MFS transporters, including five sugar transporters. The differential expression of these sugar transporters might be caused by interference of cyclosporine in glucose metabolism^96,97^. On the other hand, the majority of the remaining differentially expressed MFS genes showed similarity with members of the anion/cation superfamily that can transport a variety of substrates^98^. Finally, it should be mentioned that CsA inhibition of ABC transporters is not well characterized in arthropods compared to vertebrates^27^, and the transcriptional changes that were observed might not reflect those that are caused by the inhibition of ABC transporters but rather by inhibition of another target like calcineurin (the *T. urticae* subunits of this target are tetur04g07540, tetur04g07560 and tetur03g06410 as assessed by a blastp search, E-values between E-86 and 0 with human calcineurin subunits as queries), as occurs in the vertebrate immune system^99^.

To conclude, transcriptional changes in arthropod pests exposed to synergist compounds have only been marginally studied. In this work, we exposed a polyphagous arthropod pest, *T. urticae*, for 24 hours to four different synergist compounds - PBO, DEF, DEM and CsA - and measured genome-wide gene expression changes. The CsA treatment resulted primarily in the downregulation of *T. urticae* MFS genes, while *T. urticae* DEGs of the DEM treatment were not significantly enriched for a GO term. Exposure to PBO and DEF resulted in a broad transcriptional response and about one third of the DEGs, including cytochrome P450 monooxygenases and UDP-glycosyltransferases, were shared between both treatments, suggesting modulation of a common transcriptional program. Moreover, both DEF and PBO induced genes that are strongly implicated in acaricide resistance of the strain used in this study. Based on previous synergism toxicity studies, however, the induction of these detoxification genes seems not to interfere with the outcome of synergism assays, although the effects of induction might be relatively more important when resistance is not synergized.

## Methods

### Mite strains and chemicals

The JP-R strain has previously been described by Khalighi *et al*.^64^. Briefly, this strain is resistant to both cyenopyrafen (LC_50_ of 291 mg L^−1^) and cyflumetofen (LC_50_ of 146 mg L^−1^). In addition, cyenopyrafen toxicity in the JP-R strain is synergized by PBO and DEF, while DEM synergizes cyflumetofen toxicity^64^. The JP-R strain was maintained on potted bean plants, *Phaseolus vulgaris* L. var. Prelude, sprayed with 200 mg a.i. cyenopyrafen L^−1^ (STARMITE, 30% SC) until run-off. For the week prior to collection of RNA, the strain was maintained on bean plants without cyenopyrafen selection pressure. The synergists DEF (97% purity), DEM (97% purity) and PBO (90% purity) were of analytical grade and purchased from Sigma-Aldrich (Belgium). CsA had a purity of more than 98% and was purchased from Enzo Life Sciences (Belgium).

### Synergist bioassays

The synergist bioassays were performed as described earlier by Van Pottelberge *et al*. 2009. Briefly, synergists were dissolved in a mixture of N,N-dimethylformamide and emulsifier W (alkylarylpolyglycolether), 3:1 w/w, respectively, and subsequently diluted with deionized water 100-fold. The concentrations used for PBO (1000 ppm), DEF (500 ppm) and DEM (2000 ppm) were identical to those in Khalighi *et al*. and Van Pottelberge *et al*.^53,56^ and are known to cause between 5 and 10% mortality. Based on preliminary experiments a concentration of 50 ppm CsA was used, resulting in maximum 5 to 10% mortality. About 30 3–5 day old adult females were transferred to the upper (adaxial) side of a 9 cm^2^ square-cut kidney bean leaf discs placed on wet cotton wool. Leaf discs were sprayed at 1 bar pressure in a Cornelis spray tower with 650 µl spray fluid (1.56 ± 0.04 mg fluid deposit cm^−2^) containing one of the synergists (DEF, DEM, PBO, CsA) or formulation (FORM; N,N-dimethylformamide and emulsifier W (3:1 w/w) diluted in deionized water 100-fold) only. Unsprayed mites (CON) served as an additional control. About 600 mites (20 leaf disks with mites) were used for each treatment (DEF, DEM, PBO or CsA) and for the controls (FORM, CON). Next, leaf disks were placed in a climatically controlled room at 26 °C, 60% RH with a 16:8 h light:dark photoperiod. After 24 hours, living mites were scored and collected for RNA extraction. Mites were scored as being alive if they could walk normally after being prodded with a camel’s hair brush.

### RNA-seq

Total RNA was extracted from about 100 adult female mites (collected from at least four different leaf disks) using the RNeasy mini kit (Qiagen, Belgium) with four-fold biological replication for each treatment (PBO, DEF, DEM, CsA) and the controls (FORM, CON). The quality and quantity of the total RNA was analyzed by a DeNovix DS-11 spectrophotometer (DeNovix, USA) and by running an aliquot on a 1% agarose gel. From the RNA samples, Illumina libraries were constructed with the TruSeq Stranded mRNA Library Preparation Kit with polyA selection (Illumina, USA), and the resulting libraries were sequenced on an Illumina HiSeq 2500 to generate strand-specific paired reads of 2 × 125 bp (library construction and sequencing was performed at the High-Throughput Genomics Core of the Huntsman Cancer Institute, University of Utah, Utah, USA). Prior to read-mapping, the quality of the reads was verified using FASTQC version 0.11.4^100^ (no reads flagged as poor quality or as containing adapter sequences were used in downstream analyses).

### Expression quantification and principal component analysis (PCA)

All reads were aligned to the *T. urticae* genome^62^ using the two-pass alignment mode of STAR 2.5.0a^101^ with a maximum intron size set to 20 kb. Resulting BAM files were subsequently sorted on read name by using SAMtools 0.1.19^102^. Read counts per gene using the most recent *T. urticae* annotation (version of June 23, 2016) were then obtained using the default settings of HTSeq. 0.6.0^103^ with the “STRANDED” flag set to “yes” and the “feature” flag set to “exon”. A PCA was created as described by Love *et al*.^104^. Briefly, read counts were first normalized using the regularized-logarithm (rlog) transformation implemented in the DESeq2 (version 1.12.2) R-package^105^. A PCA was then performed using the stats (version 3.3.0) and ggplot2 (version 2.2.0) R-packages with the 5000 most variable genes across all RNA-seq samples.

### Differential expression (DE) analysis and gene ontology (GO) enrichment analysis

A differential expression (DE) analysis was performed using DESeq2 (version 1.12.2)^105^ and the read count data obtained by HTSeq (see above). Differentially expressed genes (DEGs, fold change (FC) ≥ 1.5 and Benjamini-Hochberg^106^ adjusted p-value < 0.05) were determined between unsprayed mites (CON), mites treated with DEF, DEM, PBO or CsA and mites treated with formulation (FORM) (five DE comparisons in total: FORM vs. CON, DEF vs. FORM, DEM vs. FORM, PBO vs. FORM and CsA vs. FORM). For the GO enrichment analysis, the complete *T. urticae* proteome (19087 sequences, version of June 23, 2016) was first used as query in a blastp search against the non-redundant protein database in NCBI (version of October 31, 2016) using the following settings “ -outfmt 5 -evalue 1e-5 -word_size 3 -show_gis -num_alignments 20 -max_hsps_per_subject 20”. The resulting blastp output was then loaded into the Blast2GO (version 4.0.7) program^107^ and *T. urticae* proteins were annotated using the default parameters^107^. InterProScan 5 and ANNEX were used to augment the annotation of GO terms. GO terms were condensed using the generic GO Slim subset. For the DESeq2 output of four DE comparisons (DEF vs. FORM, DEM vs. FORM, PBO vs. FORM and CsA vs. FORM), a GO enrichment analysis was performed using the Bioconductor package GOSeq (version 1.24.0)^108^, which explicitly takes into account gene selection bias due to differences in transcript length^109^. The resulting p-values from GOSeq were corrected using the Benjamini-Hochberg method^106^ and only those GO categories with an adjusted p-value of less than 0.05 were considered significantly enriched. Gene expression heatmaps were generated using the relative transcript levels (fold changes) of four DE comparisons (DEF vs. FORM, DEM vs. FORM, PBO vs. FORM and CsA vs. FORM) and the limma (version 3.28.21) and gplots (version 3.0.1) packages in the R environment. Transcription factor, P450 and UGT gene lists were obtained from previous studies^62,78,110^ while the MFS gene list consisted of those from Dermauw *et al*.^44^ and those that were differentially expressed in the CsA treatment (Supplementary Table S6). Genes with no read counts in all four DE comparison were not included in the heatplot. Finally, genes were clustered using a Euclidean distance metric and Ward’s method.

### qPCR

To validate the RNA-seq results, gene specific primers were designed for differentially expressed *T. urticae* genes (8 up- and 5 downregulated genes) using Primer 3 v.4.0.0^111^. The qPCR primers used, including the primers for the genes of interest, as well as those for the two reference genes, ribosomal protein gene RP49 and ubiquitin, can be found in Supplementary Table S8. Total RNA was extracted as described above and cDNA was synthesized with the Maxima First Strand cDNA kit (Fermentas Life Sciences, Aalst, Belgium) and 1.5 µg of total RNA. Three biological and two technical replicates were used and non-template controls were added to exclude sample contamination. The qPCR analysis was performed on a Mx3005 P qPCR thermal cycler (Stratagene, Agilent Technologies, Diegem, Belgium) with Maxima SYBR Green qPCR Master Mix and ROX solution (Fermentas Life Sciences) according to the manufacturer’s instructions. The run conditions were as follows: 95 °C for 10 m followed by 35 cycles of 95 °C for 15 s, 55 °C for 30 s, 72 °C for 30 s. At the end of these cycles, a melting curve was generated (from 65 °C to 95 °C, 1 °C per 2 s) to check the presence of a single amplicon. Fourfold dilution series of pooled cDNA were used to determine the standard curves and amplification efficiencies for every gene-specific primer pair. The efficiencies were incorporated in the calculations of the expression values. Relative expression levels and significant gene expression differences (one-sided unpaired t-test) were calculated with qbase+ version 3.0^112^.

### Image processing

CorelDRAW Home & Student ×7 and SigmaPlot 12.0 software was used for processing of images.

### Data availability

The RNA-seq expression data generated during the current study are available in the Gene-Expression Omnibus (GEO) repository with accession number  GSE98293.

## Electronic supplementary material

Supplementary Information

Table S1–S7
