# Supplementary material for: The effect of insecticide synergist treatment on genome-wide gene expression in a polyphagous pest
Source: Sci Rep. 2017 Oct 18;7:13440. doi: 10.1038/s41598-017-13397-x (PMC5647426; doi:10.1038/s41598-017-13397-x)
Supplement: Supplementary file 1 — Supplementary Information [file 41598_2017_13397_MOESM1_ESM.pdf]

Supplementary information

The effect of insecticide synergist treatment on genome-wide gene expression in a polyphagous pest

Simon Snoeck[1], Robert Greenhalgh[2], Luc Tirry[1], Richard M. Clark[2,3], Thomas Van Leeuwen[1,4,*] and Wannes Dermauw[1,*]

Affiliation

[1] Laboratory of Agrozoology, Department of Crop Protection, Faculty of Bioscience Engineering, Ghent University, Coupure links 653, 9000 Ghent, Belgium

[2] Department of Biology, University of Utah, 257 South 1400 East, Salt Lake City, Utah 84112, USA

[3] Center for Cell and Genome Science, University of Utah, 257 South 1400 East, Salt Lake City, Utah 84112, USA

[4] Institute for Biodiversity and Ecosystem Dynamics (IBED), University of Amsterdam (UvA), Science Park 904, 1908 XH, Amsterdam, The Netherlands

*corresponding authors:

thomas.vanleeuwen@ugent.be

wannes.dermauw@ugent.be

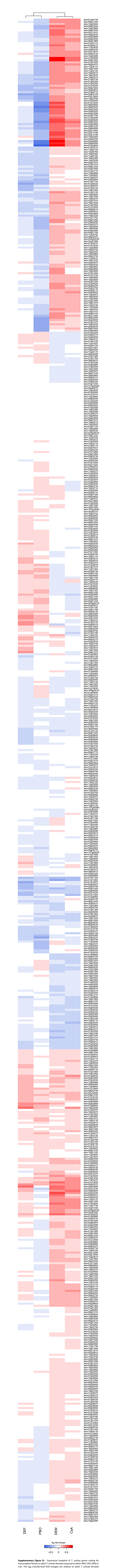

Supplementary Figure S1 - Expression heatplot of *T. urticae* genes coding for transcription factors in adult *T. urticae* females exposed to either PBO, DEF, DEM or CsA. The log_2 transformed fold changes are relative to adult *T. urticae* females treated with formulation only and were clustered using the Euclidean distance metric and Ward's method. *T. urticae* gene IDs are shown on the right. Transcription factor genes with a fold change equal to zero in at least three out of four treatments were excluded from the heatplot.

Supplementary Table S1 - Number of strand-specific paired-end Illumina reads and their mapping rates against the *T. urticae* annotation for all treatments (CON, DEF, DEM, CsA, PBO and FORM).

Supplementary Table S2 - Differentially expressed genes (absolute fold change (FC) ≥ 1.5 and Benjamini-Hochberg adjusted p-value < 0.05) between adult *T. urticae* females of the JP-R strain treated with PBO compared to adult *T. urticae* females treated with formulation only.

Supplementary Table S3 - Differentially expressed genes (absolute fold change (FC) ≥ 1.5 and Benjamini-Hochberg adjusted p-value < 0.05) between adult *T. urticae* females of the JP-R strain treated with DEF compared to adult *T. urticae* females treated with formulation only.

Supplementary Table S4 - Differentially expressed genes (absolute fold change (FC) ≥ 1.5 and Benjamini-Hochberg adjusted p-value < 0.05) between adult *T. urticae* females of the JP-R strain treated with DEM compared to adult *T. urticae* females treated with formulation only.

Supplementary Table S5 - Differentially expressed genes (absolute fold change (FC) ≥ 1.5 and Benjamini-Hochberg adjusted p-value < 0.05) between adult *T. urticae* females of the JP-R strain treated with CsA compared to adult *T. urticae* females treated with formulation only.

Supplementary Table S6 - Differentially expressed Major Facilitator Superfamily (MFS) genes (absolute fold change (FC) ≥ 1.5 and Benjamini-Hochberg adjusted p-value < 0.05) between adult *T. urticae* females of the JP-R strain treated with CsA compared to adult *T. urticae* females treated with formulation only.

Supplementary Table S7 - *T. urticae* genes coding for transcription factors with an absolute fold change ≥ 1.25 in both the PBO and DEF treatment and with an opposite direction of fold change compared to the CsA and DEM treatment.

Supplementary Table S8 - Primers used in this study.

Primer	Sequence (5'-3')	Tm(°C)	Reference	Description
tetur06g04520_F	AAATACCGAGGTCGGACGTA	59.45	(1)	Cytochrome P450 - CYP392A16
tetur06g04520_R	AAGCACTTTTTCAATCTGGTCAC	59.69	(1)	
tetur13g04550_F	CTGGCAAGCCAATGCTTTA	59.96	(1)	Intradiol ring-cleavage dioxygenase
tetur13g04550_R	ACCTCTGAGGAATCTTTCACCA	60.11	(1)	
tetur01g02650_F	CCGTCAATGTGTTAATTCAGCA	58.58	This study	Immunoglobulin E-set
tetur01g02650_R	TGTACGGAGACAGTTGGGTT	58.58	This study	
tetur09g06670_F	ATTTGATCATGGAACTGTCGGT	58.04	This study	Protein midA homolog; mitochondrial precursor
tetur09g06670_R	CCAGTGCATCATTGAGAGAAGT	58.39	This study	
tetur03g00830_F	TTGCCTGGTGGTGGTGAA	58.99	(2)	Cytochrome P450 - CYP392A12
tetur03g00830_R	ATGGTACTCTTTCCAAGACCGATA	59.04	(2)	
tetur03g00970_F	TTGTTCAATTTAGCCAAGGTGTT	57.39	(2)	Cytochrome P450 - CYP392A11
tetur03g00970_R	CCACTTTGGTTTGCTTCTCTC	57.37	(2)	
tetur11g05010-20_F	CAGTGCCAAGGAAAATGCTG	57.93	This study	Hypothetical protein
tetur11g05010-20_R	TGCGAACAATCAGTCCCAG	57.77	This study	
tetur40g00030_F	GTTACATGATTGGCGGAGTAGT	58.21	This study	Major facilitator superfamily transporter
tetur40g00030_R	GCATAGATTGGATTGAATGTGGTG	58.47	This study	
tetur08g06890_F	TGGATTATTGGGTGCTTTGACT	57.95	This study	Solute carrier family 17
tetur08g06890_R	GCAAAGTACCAATGAGATAAAATACAG	57.28	This study	
tetur08g06870_F	GTGGACTATTGGCTGCTTTATGT	59.05	This study	

Primer	Sequence (5'-3')	Tm(°C)	Reference	Description
tetur08g06870_R	TGATAACGAGTTGAAAAGTGCCT	58.61	This study	Sodium-dependent phosphate transporter
tetur06g01700_F	CTAATGATTCACGACCATGACACT	58.89	This study	Glycoside hydrolase
tetur06g01700_R	CAGCTCATCATAATAGTGACCGAA	58.47	This study	
RP49_F	CTTCAAGCGGCATCAGAGC	62.19	(1)	Ribosomal protein 49
RP49_R	CGCATCTGACCCTTGAACTTC	62.09	(1)	
UBQ_F	GTCTCCGTGGTGGAATGC	58.11	(3)	Ubiquitin C
UBQ_R	TTGGATTTTGGCTTTCACG	54.50	(3)	

(1) Dermauw, W. *et al.* A link between host plant adaptation and pesticide resistance in the polyphagous spider mite *Tetranychus urticae*. *Proc. Natl. Acad. Sci. U. S. A.* **110,** E113-22 (2013).

(2) Khalighi, M. *et al.* Molecular analysis of cyenopyrafen resistance in the two-spotted spider mite *Tetranychus urticae*. *Pest Manag. Sci.* **72,** 103–112 (2015).

(3) Demaeght, P. *et al.* Molecular analysis of resistance to acaricidal spirocyclic tetronic acids in *Tetranychus urticae*: CYP392E10 metabolizes spirodiclofen, but not its corresponding enol. *Insect Biochem. Mol. Biol.* **43,** 544–554 (2013).
